# Compression-distraction reduction surgical verification and optimization to treat the basilar invagination and atlantoaxial dislocation: a finite element analysis

**DOI:** 10.1186/s12938-016-0246-2

**Published:** 2016-12-28

**Authors:** Xuefeng Bo, Weida Wang, Zan Chen, Zhicheng Liu

**Affiliations:** 1 School of Biomedical Engineering, Capital Medical University, Beijing, 100069 China; 2 Beijing Key Laboratory of Fundamental Research on Biomechanics in Clinical Application, Capital Medical University, Beijing, 100069 China; 3 Pera Corporation Ltd. Beijing Branch, Beijing, 100025 China; 4 Xuanwu Hospital, Capital Medical University, Beijing, 100053 China

**Keywords:** Basilar invagination, Atlantoaxial dislocation, Compression-distraction reduction technique, Surgical optimization

## Abstract

**Background:**

Basilar invagination (BI) combined with atlantoaxial dislocation (AAD) leads to foramen magnum stenosis and medullary spinal cord compression, causing nerve dysfunction. The purpose of the surgery is to remove the bony compression at brainstem ventral side and fix the unstable spinal segment and make it fused stably. Occipital cervical internal fixation system that simultaneously reduces atlantoaxial horizontal and vertical dislocation are established. We propose here a new compression-distraction reduction (CDR) technique. We aimed to construct a congenital BI-AAD preoperative finite element model (FEM) to simulate the CDR technique for AAD reduction surgery.

**Methods:**

Based on computed tomographic scans of patients’ cervical vertebrae, a three-dimensional (3D) geometric model of the cervical spine (C0–C4) of congenital BI-AAD patients was established using Mimics13.1, Geomagic2012, and Space Claim14.0 softwares. The mechanical parameters of the tissues were assigned according to their material characteristics using ANSYS Workbench 14.0 software. A 3D FEM was established using the tetrahedral mesh method. The bending moment was loaded on C0. Physiological conditions—anteflexion, retroflexion, left and right flexion, left and right rotation—were simulated for preoperative verification. The occipital cervical fixation system FEM was established. The CDR technique was simulated to perform AAD reduction surgery. Data were obtained when the atlantoaxial horizontal and vertical dislocation reductions were verified postoperatively. Stress data for the two surgical schemes were analyzed, as was the reduction surgery optimization scheme for congenital BI-AAD patients with abnormal lateral atlantoaxial articulation ossification.

**Results:**

Cervical spine (C0–C4) FEM of congenital BI-AAD patients was established. The CDR technique was simulated for AAD reduction. We obtained the mechanical data when the atlantoaxial horizontal and vertical dislocation reductions were satisfied for the two surgical schemes.

**Conclusions:**

The CDR technique for AAD reduction was feasible and effective. We propose this reduction optimization scheme for patients with lateral atlantoaxial articulation due to abnormal ossification of congenital BI-AAD. We also provide a biomechanically theoretical basis for improving the reliability of pure posterior reduction surgery and simplifying surgery for complicated BI-AAD disease.

## Background

Basilar invagination is a common clinical bone malformation at the craniocervical junction. It mainly manifests as fusion of the atlas and occipital bone, fusion of the occipital condyle and atlas lateral bone, and loss in height of the lateral atlantoaxial articulation. The dentoid process of the axis moves and protrudes into the foramen magnum, with lateral atlantoaxial articulation and malformation of the dentoid process of the axis after fusion of the atlas and occipital bone. The odontoid process dislocates upward and backward, which may compress the medulla oblongata and upper cervical spinal cord, resulting severe neurological dysfunction and even paralysis [1].

The primary purpose of surgical treatment for complicated basilar invagination and atlantoaxial dislocation is to remove the ventral bony compression of the brainstem. The secondary purpose is to fix the unstable spinal segment and achieve bone fusion and stability. The past main treatment strategy was preoperative or intraoperative skull traction and cervical radiographic observation. For atlantoaxial dislocation, which can be reduced, posterior occipital cervical internal fixation fusion surgery can be performed in the case of atlantoaxial reduction by skull traction. For atlantoaxial dislocation, which cannot be reduced, the current main belief is that longitudinal traction cannot achieve atlantoaxial reduction because of contracture of various ligaments between the atlantoaxial vertebrae and scar formation between the anterior arch of the atlas and the dentoid process of the axis. Therefore, the transoral approach should be adopted for atlantoaxial anterior release. Then, after reducing the atlantoaxial dislocation by skull traction, posterior occipital cervical fixation fusion surgery [2–5] should be carried out. Because odontoid surgical trauma is extensive due to the incision and removal or release, as well as the high incidence of complications (e.g., surgical infection, breathing difficulty, swallowing difficulty), the patient should not be subjected to suffering greater risk and pain. The clinical treatment is gradually changing from conventional anterior release, traction, reduction, and posterior fusion to a simpler posterior cutting-reduction-fixation technique.

**Fig. 1:**
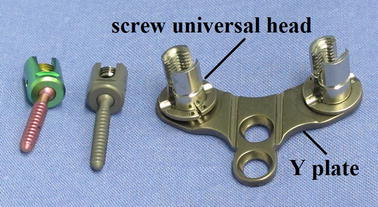
Compression occipital plate (COP)

In 2010, Jian et al. [6, 7] reported the use of a distraction technique between posterior screws for atlantoaxial dislocation reduction, with good results. The great distraction force between the occipital screw and inter-odontoid vertebral arch pedicle and the vertical dislocation between atlantoaxial sites could be effectively reduced, although the technique was still inadequate (i.e., the mechanical property for directly reducing the atlantoaxial horizontal dislocation was lacking). Chen et al., at Xuanwu Hospital, proposed a new compression-distraction reduction (CDR) technique. After long-term clinical practice, they designed an occipital cervical internal fixation system that could reduce the atlantoaxial horizontal and vertical dislocations simultaneously. The core was the compression occipital plate (COP) (Fig. 1). Good results were achieved after a preliminary clinical application, combining it with a simple posterior cutting reduction and fixation technique.

Basilar invagination is often accompanied by complex bone malformation at the craniocervical junction. In addition, the atlanto-odontoid joint between the atlantoaxial sites and the joints on both sides may also have suffered complex malformation. Some scholars [8] established a three-dimensional (3D) model of lateral atlantoaxial articulation (LAA) in congenital abnormal ossification using thin-slice computed tomography (CT) data from 63 patients, with 20 patients in the control group. They divided the LAA abnormalities into four categories according to the degree of spondylolysis and the atlantoaxial joint surface inclination angle. Using morphological measurement data, the incidence of atlantoaxial dislocation increased from type I to type I, and the basilar invagination degree gradually increased from type I to type III. Among them, surgery should be carried out for types II, III and IV. Therefore, for research and treatment, attention should be paid not only to whether the atlantodental gap is increased but to the impact of lateral atlantoaxial articulation during the reduction surgery. Changes in the latter’s anatomy and biomechanics are particularly important for developing a surgical scheme.

Biomechanical studies on the spine are generally performed on cadaver specimens, animal models, and/or on an FEM. The rare complex 3D malformations that are characteristic in patients with congenital BI makes such cadavers scarce. In addition, there are no appropriate animal models as yet [9–11]. Therefore, the present study used an FEM for simulation of the calculation and analysis. We constructed a congenital basilar invagination atlantoaxial dislocation (BI-AAD) FEM and an occipital cervical internal fixation system FEM for our two surgical schemes. Then, we simulated AAD reduction surgery with a CDR technique. Later, we analyzed the mechanical data acquired when the atlantoaxial horizontal dislocation and vertical dislocation were satisfied to confirm the feasibility and effectiveness of the surgical technique. Finally, we researched the reduction surgery optimization scheme for patients with congenital BI-AAD with lateral atlantoaxial articulation and abnormal ossification (upper and lower joint surfaces had completely slipped or were completely separated).

## Methods

### Materials, hardware configuration and modeling software

Volunteers with lateral atlantoaxial articulation abnormalities (type III) along with congenital BI-AAD, including basilar impression, atlantoaxial dislocation, atlanto-occipital partial fusion, and cervical vertebral C2–C3 fusion were studied. CT was performed on the patient’s cervical spine. The patients were placed in supine position, with their intercilium and nose tip coinciding with the scanning center line. The axis position was continuously scanned (scanning range C0–C7). The scanning layer distance was 0.6 mm. C0–C4 tomographic images were obtained, and the data were stored in 512 × 512 pixel Digital Imaging and Communication in Medicine format. The following equipments were used in this study: Siemens SOMATOM Sensation 64/Cardiac 64 spiral CT machine; Dell Precision T7610 workstation; Mimics 13.1, Geomagic 2012, and ANSYS14.0 software.

### FEM of C0–C4 segments in congenital BI-AAD: construction and verification

**Fig. 2:**
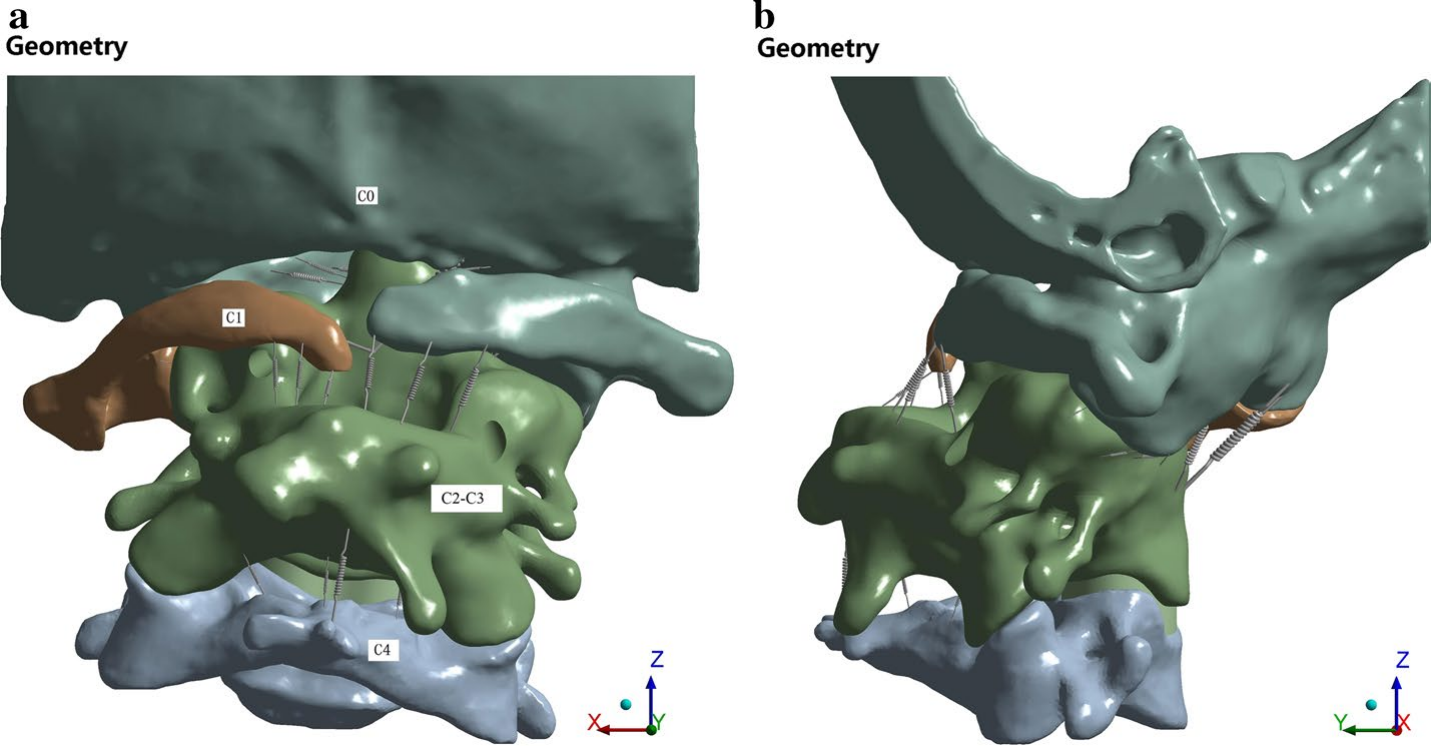
Three-dimensional geometric model of the cervical spine of the C0–C4 segments. **a** Preoperative geometric model (*front view*). **b** Preoperative geometric model (*side view*)

The scanned images were imported into Mimics 13.1 software. The vertebral contour was extracted for 3D surface reconstruction. Sufficient C0 and COP constraint parts were retained to reduce the computing cost of the finite element simulation. The 3D image data for the C0–C4 vertebrae were saved in .stl format. We used Geomagic 2012 for non-continuous point cloud and noise point processing and for generating curved surface segments. The point cloud was then packaged into a polygon after deleting the non-connection point cloud and isolated point. We eliminated the noise and performed other processing operations at the point stage. After simplifying, hole filling, characteristic removing, sand-papering, repairing the intersection area, and performing other processing operations at the polygon stage, the 3D geometric model of C0–C4 vertebrae were saved in .igs format. In accordance with the clinical anatomy illustrations and clinical nuclear magnetic resonance images, the cartilage was created in ANSYS SpaceClaim. The minimum gaps of mutual friction on the bone of C0–C1 and C1–C2 were 0.13 and 0.26 mm, respectively. The corresponding thicknesses of the joint cartilage were 0.065 and 0.13 mm, respectively. After C2–C3 fusion, the intervertebral disc model was established only between C3 and C4. In according with the clinical experience and ligament anatomical images, the attachment points of the ligament were determined on the vertebral body model, and the ligament was created with a spring unit. A 3D geometric model of cervical spine segments C0–C4 was obtained, as shown in Fig. 2.

In this study, the vertebrae were treated as isotropic linear elastic material. Because the material properties in the various parts of the model were very different, different material properties were assigned respectively [12] according to the anatomical structural characteristics of the cervical spine as shown in Table 1.

**Table 1:** Material parameters used in the finite element model

Tissue structure	Young’s modulus of elasticity	Poisson’s ratio	Thickness (mm)
Cortical bone	12,000	0.3	1
Spongy bone	250	0.29	
Intervertebral disc	500	0.45	
Cartilage C0–C1	10.4	0.4	0.065
Cartilage C1–C2	10.4	0.4	0.13

**Fig. 3:**
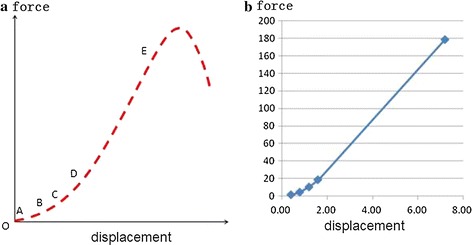
Ligament stress-displacement curve. **a** Actual stress-displacement curve. **b** Finite element spring input stress-displacement curve, such as the AP ligament

The ligament not only could bear the tension load, its rigidity was highly nonlinear (as shown in Fig. 3, stress-displacement curve). We selected the Combine14 spring unit to simulate the unidirectional nonlinear rigidity of the ligament using the segmented linear method, as shown in Fig. 3. The straight-line OA, AB, BC, CD, and DE rigidities were 10, 30, 50, 70, and 100%, respectively, of the maximum rigidity. The specific rigidity values are shown in Table 2 [13, 14]. The transverse ligament was the shell element, the intermediate portion was connected with the common node of the odontoid process, and both sides were connected with a common occipital node.

**Table 2:** Nonlinear spring input rigidity

Ligament name	Numbers		A	B	C	D	E
	Preoperative	Postoperative					
Apical ligament AP	1	1	0.40	0.80	1.20	1.60	7.20
			1.14	4.58	10.30	18.30	178.46
Alar ligament AL	8	8	0.07	1.41	2.12	2.82	12.69
			0.45	1.79	4.02	7.14	69.62
Anterior longitudinal ligament AAL	3	3	0.20	0.40	0.60	0.79	3.57
			0.10	0.41	0.91	1.62	15.82
Joint capsule JC C1–C2	16	16	0.50	0.99	1.49	1.98	8.91
			0.10	0.40	0.91	1.61	15.73
Atlantoaxial ligament AAAL	2	2	0.20	0.40	0.60	0.79	3.57
			0.10	0.41	0.91	1.62	15.82
Accessory ligament	2	2	0.20	0.40	0.60	0.79	3.57
			0.10	0.41	0.91	1.62	15.82
Tectorial membrane TM	5	5	0.60	0.08	0.60	0.08	0.60
			1.19	0.34	1.19	0.34	1.19
Anterior atlantooccipital membrane AAOM	4	4	0.95	1.89	2.84	3.78	17.01
			0.23	0.91	2.05	3.65	35.59
Ligamenta flava LF	6	4	0.48	0.96	1.44	1.92	8.64
			0.08	0.32	0.72	1.27	12.41

For preoperative verification, the model contact settings included the following. The frictionless contact model was set between the C0 joint cartilage and C1 joint cartilage, the C1 joint cartilage and C2 joint cartilage, the odontoid process and occipital hole [11]. A bonded contact was set between the intervertebral disc and the vertebrae so as not to allow relative displacement. The fixed support was applied on the bottom surface of the cervical vertebra, C4 segment, to constrain displacement of the lower vertebral body for each degree of freedom (1.8 N m). Moment was applied on the upper skull interface [9]. According to the right-hand screw rule, the torques in the corresponding sagittal, coronal, and shaft planes were generated to simulate six physiological conditions: anteflexion, retroflexion, left and right lateral bending, left and right rotation.

**Fig. 4:**
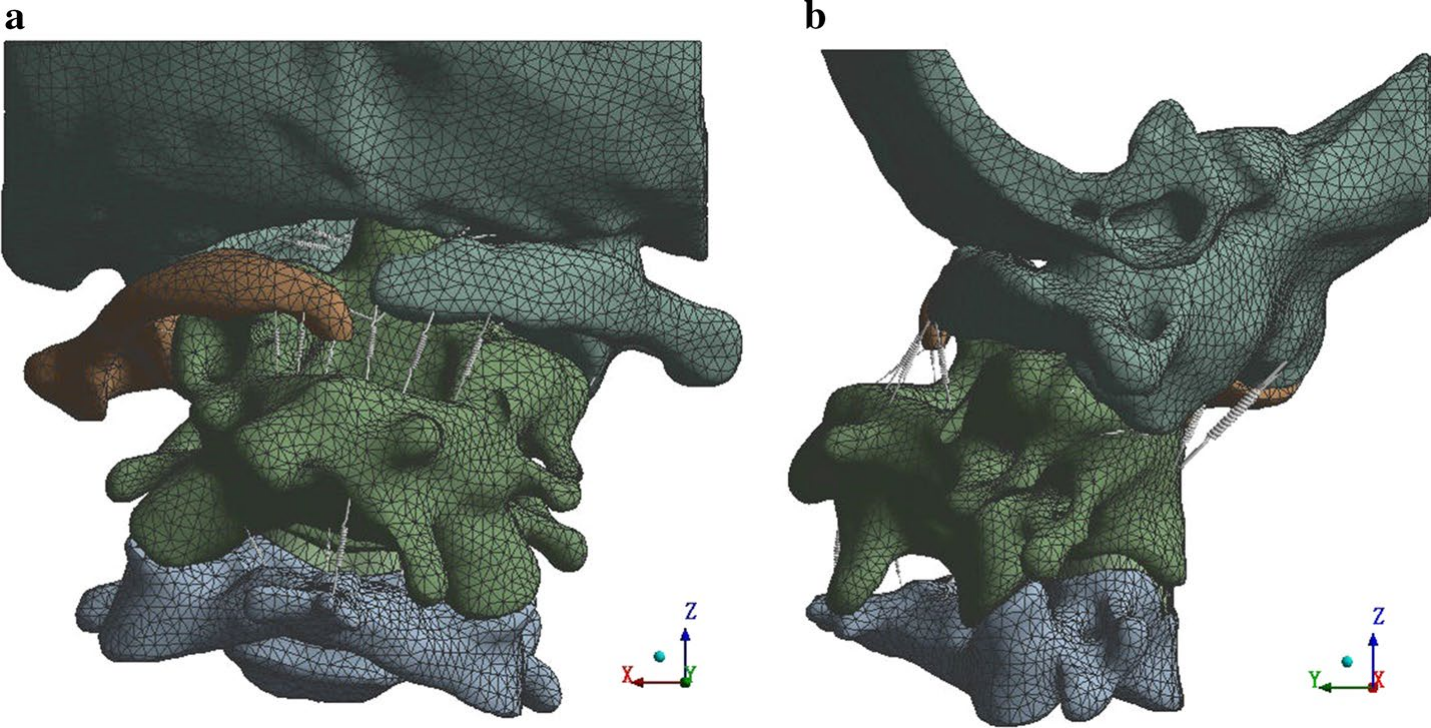
Three-dimensional finite element model (FEM) of the cervical vertebrae of the C0–C4 segments. **a** Preoperative FEM (*front view*). **b** Preoperative FEM (*side view*)

The 3D FEM of the cervical vertebra was divided based on the mesh in ANSYS 14.0 software, which included 226,522 nodes and 145,610 units. The crucial parts (e.g., C1, C2–C3, joint cartilage) were divided with a local mesh division method, with a unit size of 1.5–2.0 mm. The 3D FEM of the cervical vertebrae is shown in Fig. 4.

We then analyzed displacement and stress by combining clinical practice after preoperative model loading. The results showed that the maximum displacement in anteflexion was 9 mm, located at the upper boundary of the skull. Through a comparative analysis of the clinical data, we learned that, because the dislocation in the patients could be more serious, the range of motion might be less if there were more constraints between C1 and C2–C3. For cervical maximum angle of anteflexion and retroflexion, we compared clinical measurement data and model simulation data to illustrate the effective of the models. Consistent with clinical preoperative CT image, ROM between C3 and C4 are 13.04° (anteflexion) and 2.01° (retroflexion).

### CDR technique simulated for virtual AAD reduction surgery

**Fig. 5:**
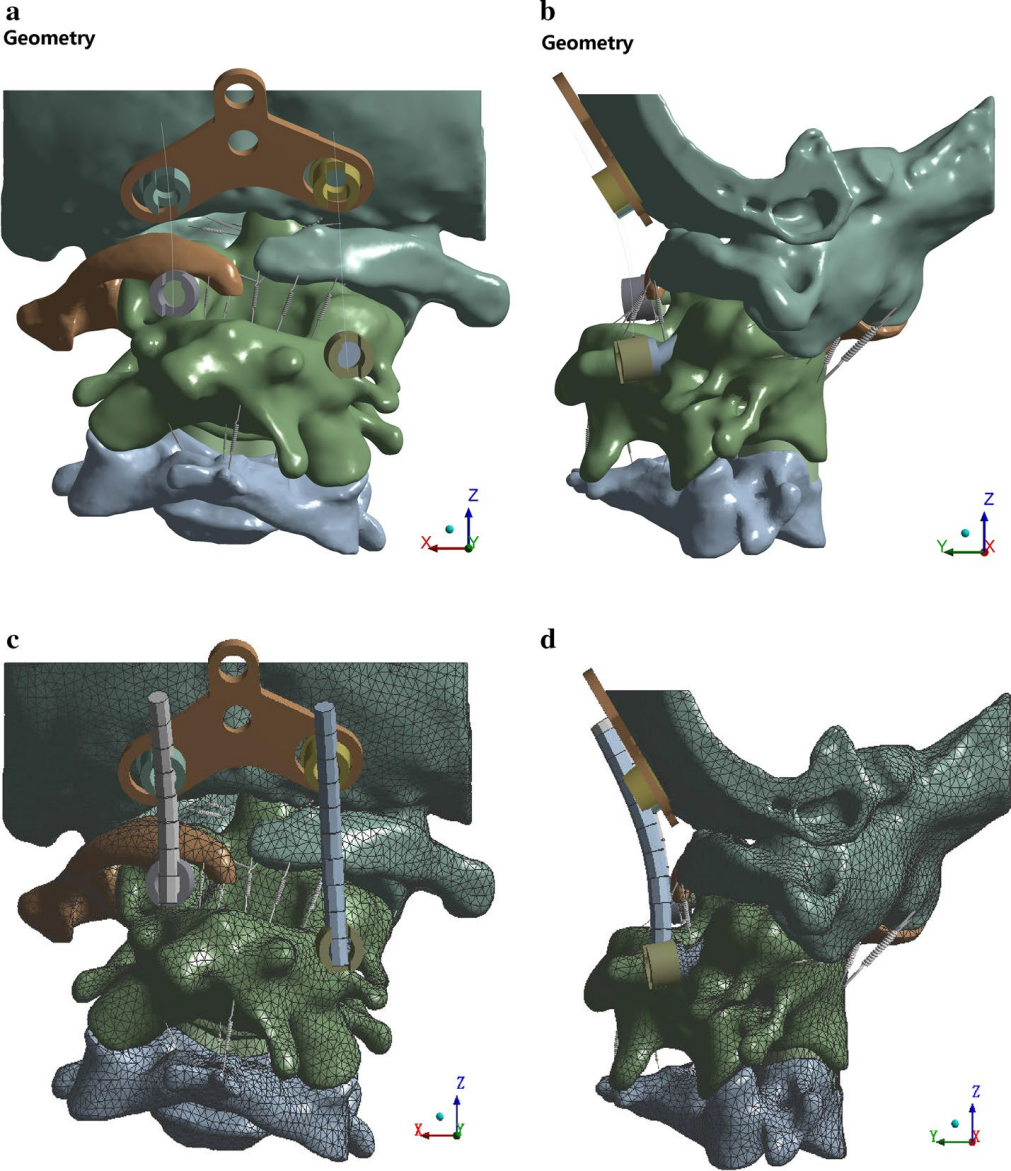
Intraoperative geometric model and FEM. **a** Operative geometric model (*front view*). **b** Operative geometric model (*side view*); **c** Operative FEM (*front view*). **d** Operative FEM (*side view*)

The titanium rod geometrical model and the positions of four pedicle screws were determined by referring to the patients’ postoperative CT image data. The unit mesh was divided in ANSYS, and the FEM was generated, as shown in Fig. 5. Material properties are shown in Table 3. The Y plate, screw universal head, and U-shaped nail were simplified as “rigid,” and the beam unit was used to simulate the titanium rod in the COP.

**Table 3:** Material parameters used in the compression occipital plate model

System structure	Young’s modulus of elasticity (MPa)	Poisson’s ratio	Brand no.
Titanium rod	114,000	0.36	TC4
EOP	Rigid body		
Screw	Rigid body		

**Fig. 6:**
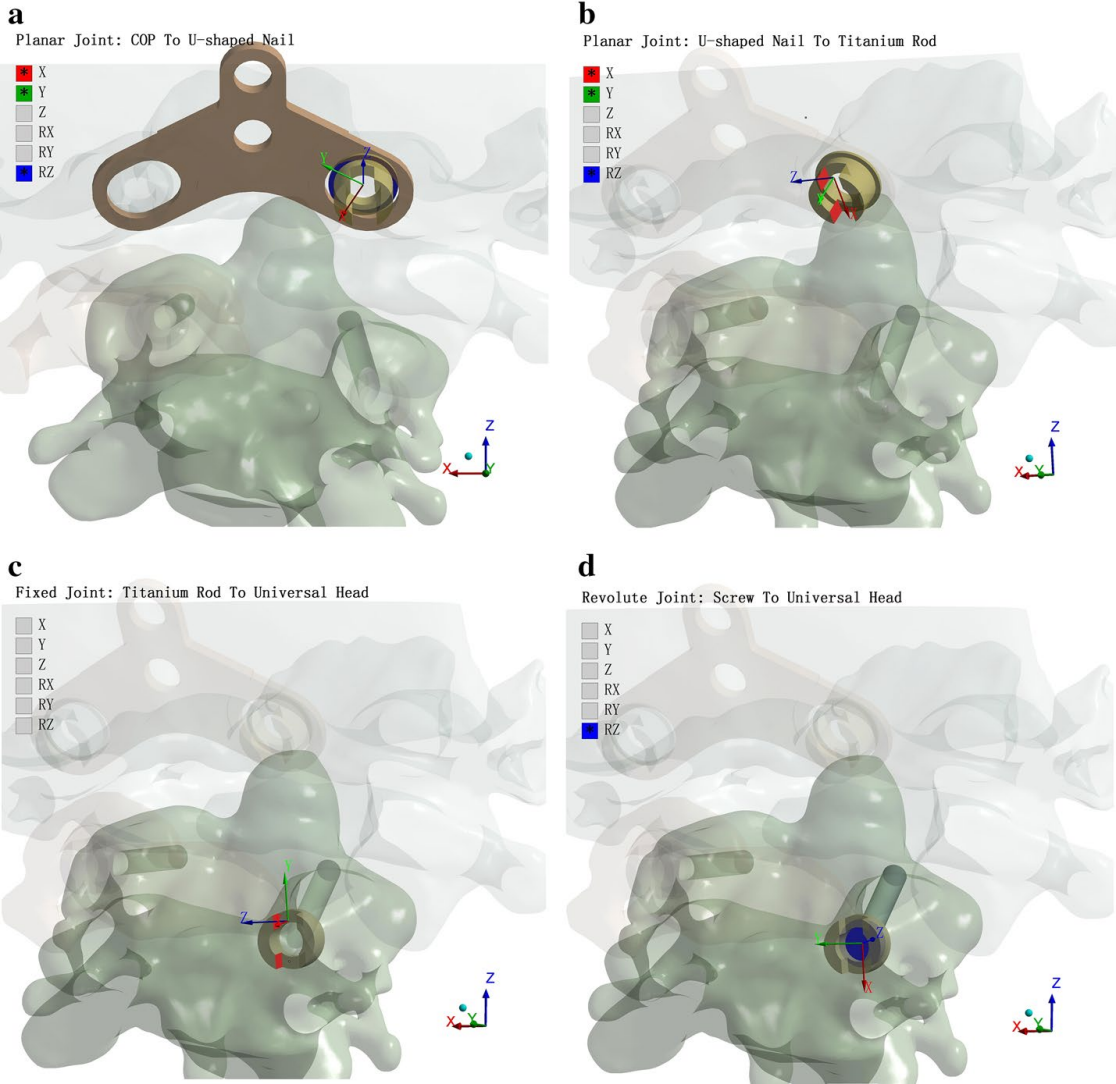
Degrees of freedom constraints of the model. **a** Planar joint COP to U-shaped nail. **b** Planar joint U-shaped nail to titanium rod. **c** Fixed joint titanium rod to universal head. **d** Revolute joint screw to universal head

According to the actual surgical process, the fixed support was applied on the skull interface to constrain the displacement. The six degrees of freedom (DOFs) were made on the COP universal screws with joint connections, as shown in Fig. 6. The COP and U-shaped nail belonged to a planar joint. The UZ, RX, and RY DOFs were constrained. The U-shaped nail could be translational and rotated within the COP long hole. The U-shaped nail and titanium rod belonged to a planar joint—i.e., corresponding to the first step (compression) and the second step (distraction) during the surgical process). As the titanium rod and universal head fixed all DOFs, relative displacement could not occur. The screw and universal head belong to a revolute joint, so the universal head could be rotated freely only along the screw’s axial direction.

The simulation process of reduction surgery was divided into two steps, which were solved sequentially, with horizontal reduction and vertical reduction. The multi-step static structure method was used. In step 1, horizontal reduction was started. A displacement load was applied to C2 to make it move in a parallel direction to accomplish satisfactory horizontal reduction. In step 2, vertical reduction was carried out. The displacement load was applied continuously to C2 to accomplish vertical displacement. The displacement distance was chosen by referring to the postoperative CT image. The vertical reduction position was confirmed with the clinician.

**Fig. 7:**
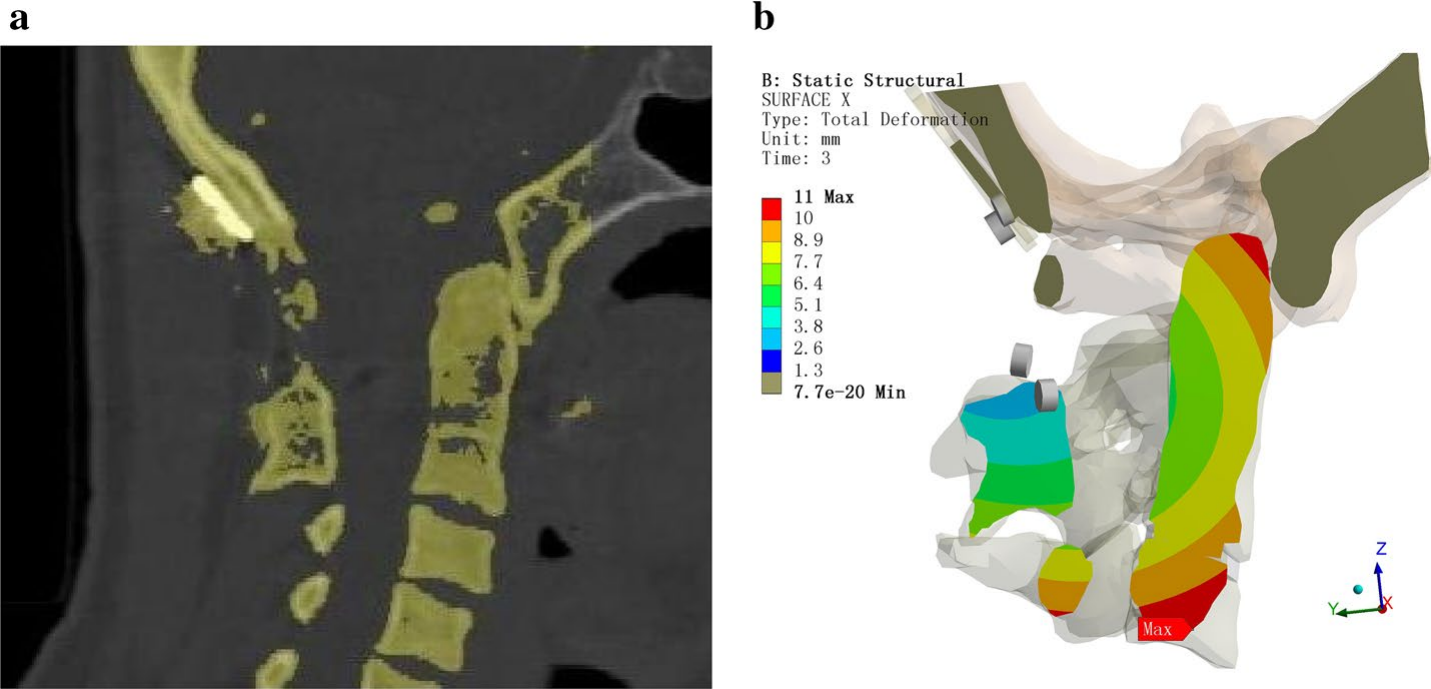
Comparison chart for the mid-sagittal plane. **a** Computed tomography (CT). **b** ANSYS displacement cloud chart

**Fig. 8:**
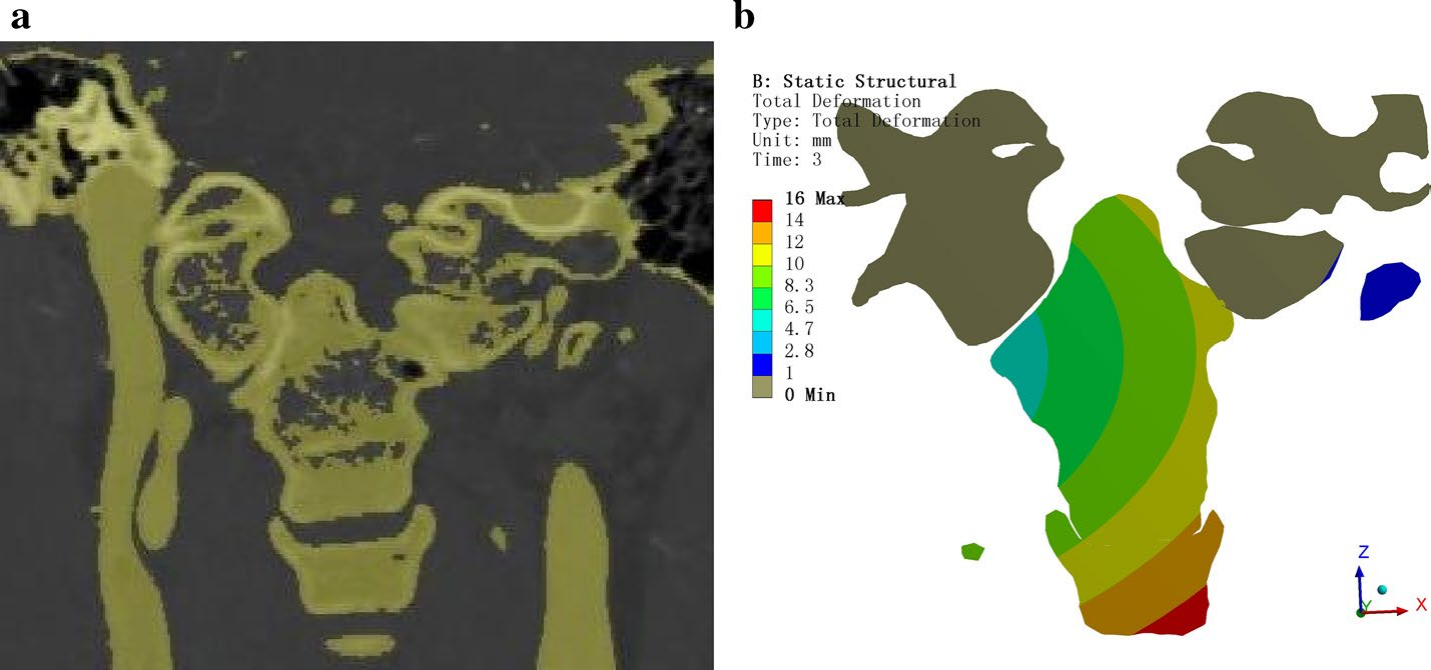
Comparison chart for the coronal plane. **a** CT. **b** ANSYS displacement cloud chart

**Fig. 9:**
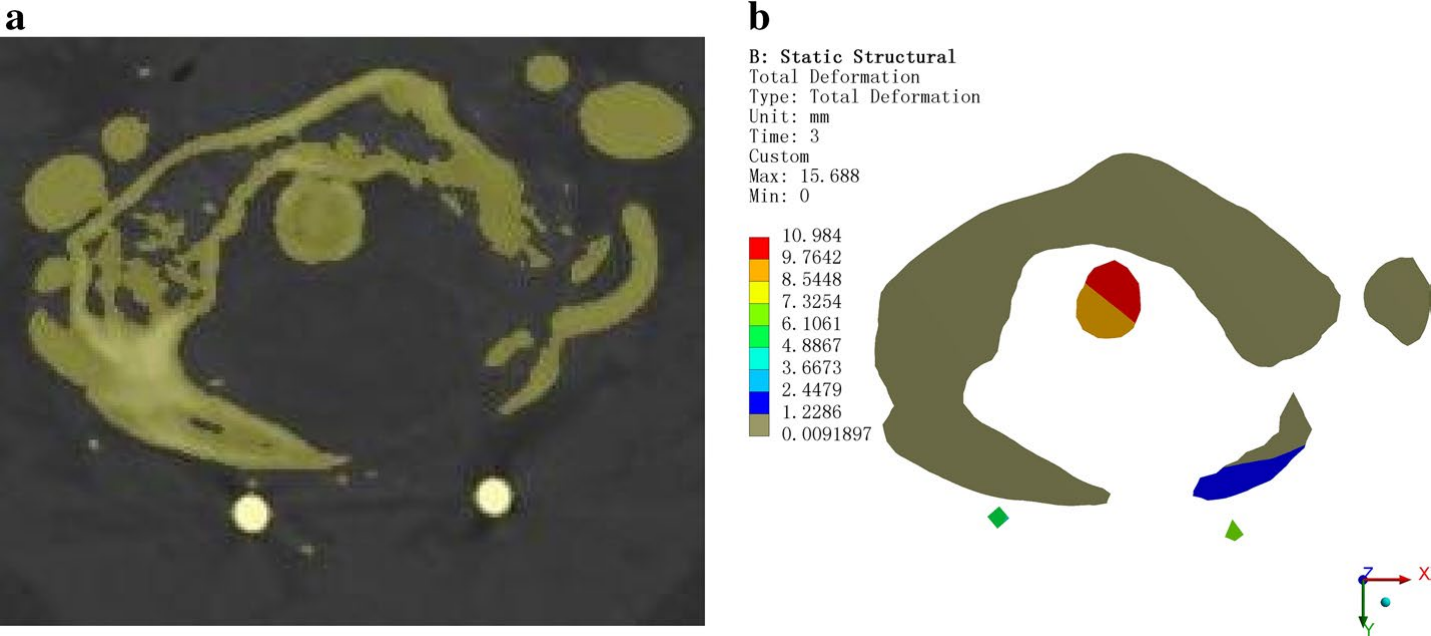
Comparison chart for the horizontal plane. **a** CT. **b** ANSYS displacement cloud chart

For postoperative verification, the vertebral positions were recorded at each step. The clinicians referred to the postoperative CT images to confirm the outcomes, as shown in Figs. 7, 8, and 9.

Through analysis of the displacement and stress after model loading and combining it with the clinical practice results, it could be seen that the degree of activity between the skull and C4 was decreased. Taking anteflexion as an example, the postoperative skull maximum displacement was 0.02 mm and the amount of displacement was mainly produced in the intervertebral discs of C3 and C4. Comparing the clinical postoperative image data of anteflexion and posterior extension, the ranges of the activities were seen to be consistent.

## Results

### Verification of the feasibility and effectiveness of CDR in AAD reduction surgery

The CT image data were used to verify the feasibility and effectiveness of the CDR technique in AAD reduction surgery. Figure 7 shows a comparison chart of the mid-sagittal plane. The odontoid process angle and position are consistent with the postoperative CT measurement data. Figure 8 is a comparison chart of the coronal plane. The odontoid process and the position of C1 are consistent with the postoperative CT measurement data, although occlusion of the odontoid process and joint surfaces on C1 is inconsistent with the postoperative CT, which might be due to the CT image data having been obtained 6 months after surgery when fusion had already occurred. Figure 9 is the horizontal surface control chart. The position of the odontoid process is consistent with the postoperative CT measurement data. All of these data show that simulation of the surgical horizontal and vertical reduction effects meet the clinical requirements of AAD reduction surgery.

The CDR technique was simulated for AAD reduction surgery on the abovementioned congenital BI-AAD patient (abnormal ossification of the lateral atlantoaxial articulation). The mechanical change in the atlantoaxial complex body was solved step by step (especially the lateral atlantoaxial articulation and surrounding ligaments). In addition, the mechanical data for AAD reduction (both horizontal and vertical dislocations) were analyzed and satisfied the clinical requirements.

**Fig. 10:**
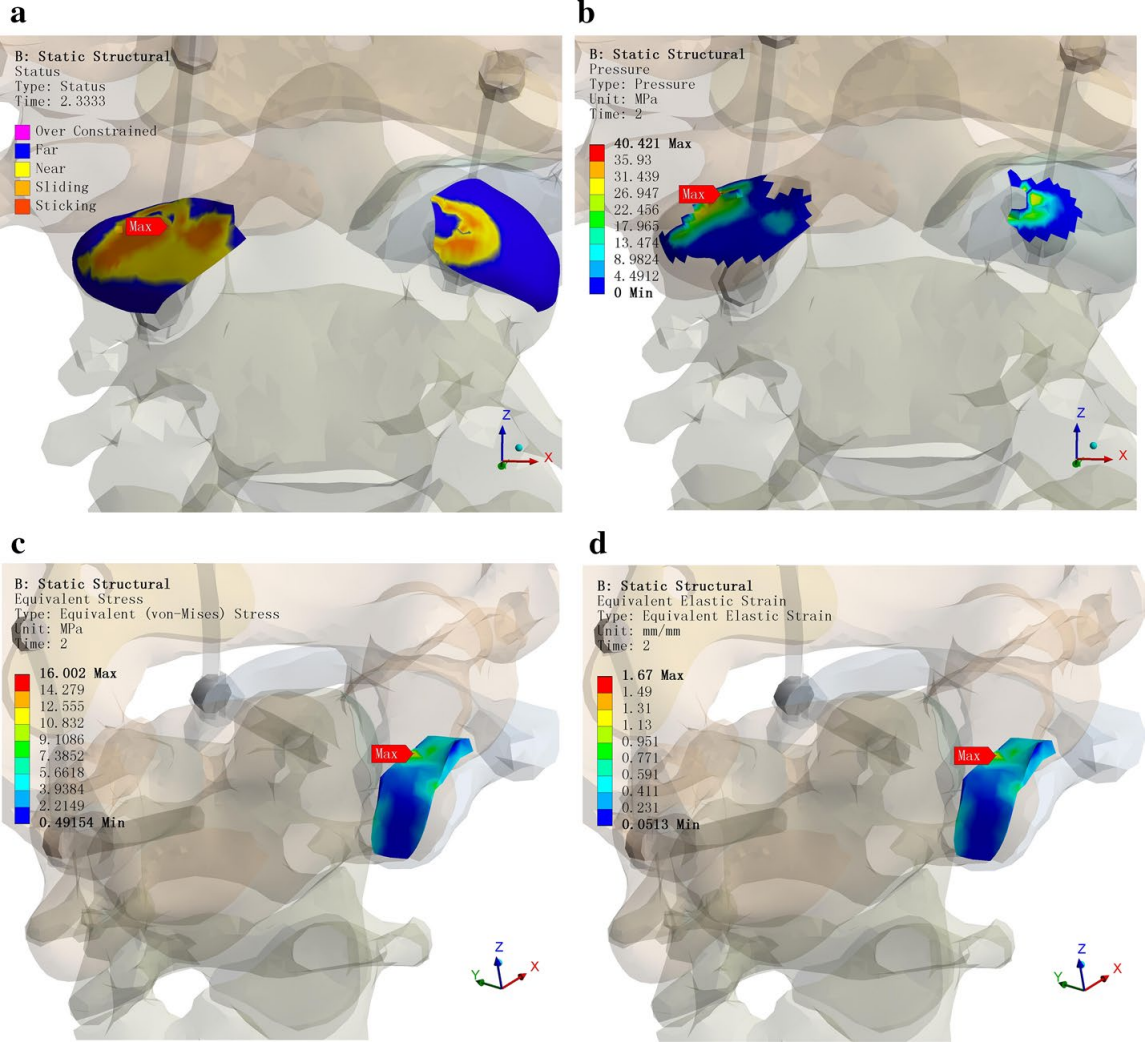
Important tissue stressing cloud chart during the surgical process. **a** C1–C2 cartilage contact state. **b** C1–C2 cartilage contact pressure. **c** C0–C1 soft tissue stress maximum value. **d** C0–C1 soft tissue strain maximum value

As shown in Fig. 10, as blockage of the C1 lateral block joint in the first step of the “compression” process, there was a trend for C1 to block odontoid process reduction, so the lateral joint cartilage of C1 and C2 fit tightly, and the pressure was relatively large. The maximum contact surface pressure was 31.98 MPa during the second step, the “distraction” process, C1 cartilage was susceptible to damage.

During the surgery, the titanium rod must be pressed into the U-shaped nail so it can be distracted by pliers. If the reduction is forced, the C1 cartilage would inevitably be damaged. If the AAD cannot be reduced, it would influence the surgical effect. Therefore, the following optimization surgery schemes were required: distraction, compression, and finally distraction again.

### Optimization surgery for reduction in a congenital BI-AAD patient (abnormal ossification of the lateral atlantoaxial articulation)

According to the malformation characteristics of the congenital BI-AAD patient (abnormal ossification of the lateral atlantoaxial articulation), the study presented an optimization surgery scheme: minor displacement (6 mm), vertical reduction, and then horizontal and vertical reduction. Similarly, the mechanical change in the atlantoaxial complex body was solved step by step (especially lateral atlantoaxial articulation and the surrounding ligaments). When AAD reductions of the horizontal and vertical dislocations were accomplished, the mechanical change was analyzed to provide a biological mechanical basis for improving the treatment scheme.

**Fig. 11:**
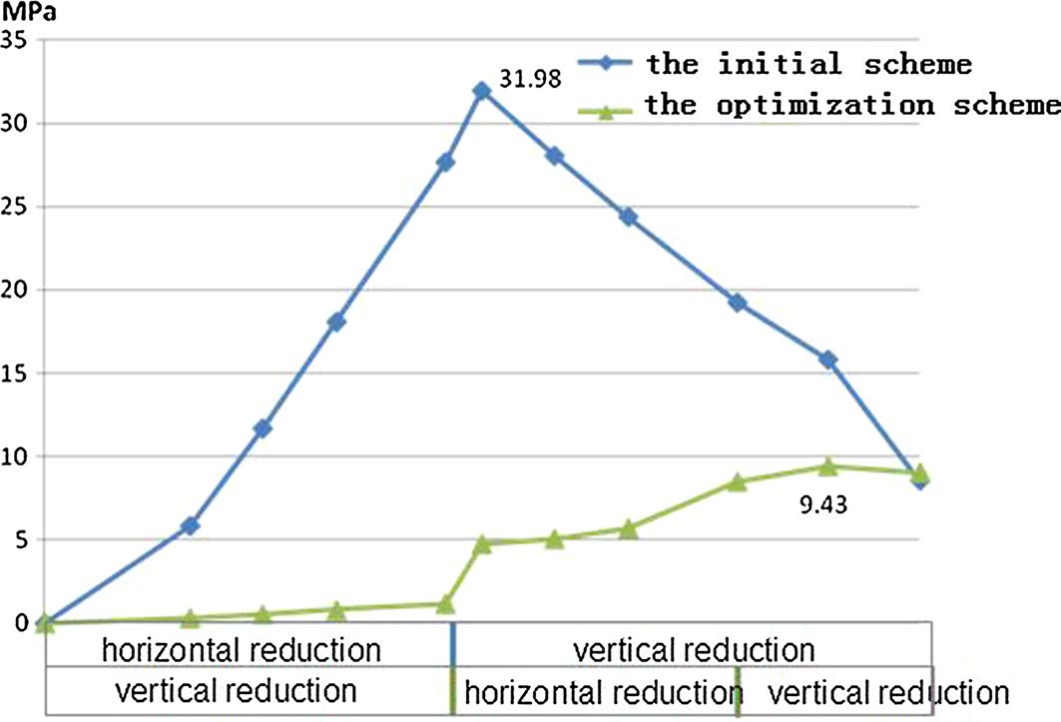
C1-C2 cartilage contact pressure comparison of the two schemes

As shown in Fig. 11, the cartilage pressure values during the surgical portions of the two surgery schemes were compared. The maximum value for the initial scheme was 31.98 MPa, which occurred when the horizontal displacement of the first step ended. The maximum value for the optimization scheme was 9.43 MPa, which occurred at the end of all reductions.

**Fig. 12:**
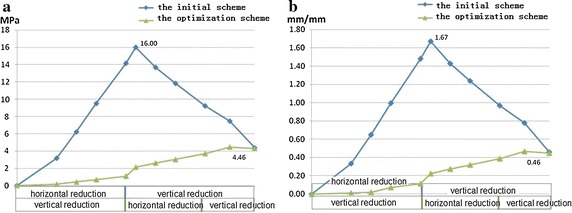
C0–C1 soft tissue stress compression of two schemes. **a** Stress value. **b** Strain value

As shown in Fig. 12, the soft tissue stress/strain values between C0 and C1 during the surgical stage of the two surgery schemes were compared. The soft tissue maximum stress/strain values for the initial scheme occurred after horizontal reduction, with the maximum stress value 16 MPa and the maximum strain value 1.67 mm/mm. The soft tissue maximum stress/strain in the optimization scheme occurred during the vertical reduction process of the second step. The maximum stress value was 4.46 MPa, and the maximum strain value was 0.46 mm/mm.

These results showed that, after using the optimization scheme, the cartilage contact pressure between C1 and C2 was alleviated, which was consistent with the clinicians’ experience.

## Discussion

Clinical studies have shown that relevant lesions of the occipital-atlanto-axial complex were mostly based on congenital bone malformations [15]. The complex geometric characteristics of the region also determined its complex biomechanical properties.

Yin et al. [16] speculated that the AAD caused by the congenital malformation was not due to atlantoaxial median joint dislocation alone. The more prominent cause was the structural change and malformation of the lateral atlantoaxial articulation, which affected the movement mode and load-bearing mode between the atlas and the atlantoaxial joint. Finally, atlantoaxial stability caused changes. These authors believed that types I and II could be reduced or partially reduced under certain conditions, but it was difficult or impossible to reduce type III, as the anterior odontoid process must be ground and posterior fixation then carried out. The surgical plan for type IV should be developed according to the presence or absence of compression on the ventral side.

Salunke et al. [17] proposed the concept of determining the angle between the straight line through the joint surface under the atlas and the straight line from the upper point to the rear edge of the hard palate in the sagittal plane. They believed that direct reduction could be carried out through a posterior surgical approach if the angle was >150°.

Because of the great individual differences in malformations of the craniocervical junction region, it is unrealistic to include all of the abnormal circumstances under the umbrella of a single surgical treatment scheme. In terms of the surgical technique via the posterior approach using mechanical force between the occipital and pedicle screws to distract them and create the distraction, there will also be a certain applicable range or personalized optimization scheme. In multi-dimensional space, the biomechanical and load-bearing point change caused by the structural variation of the lateral joint plays a clear role in guiding the clinician to the correct diagnosis when facing a complicated AAD, which, in turn, provides the basis for the development of a reasonable treatment scheme and surgical strategy. The clinical treatment choice should not only be based on the type of malformation but also on the biomechanical situation of the affected segments. Thus, one should use personalized treatment principles and design an occipital cervical internal fixation system based on personalized parameters and a reasonable treatment scheme with the aim of improving the prognosis.

In this study, simulation of BI-AAD pathological and physiological states in a congenital BI-AAD FEM not only compensated for the lack of animal models and cadaver specimens, it simulated a compression-distraction reduction technique. We thus verified the feasibility and effectiveness of AAD reduction surgery. The analysis method of multiple load steps was used. A plurality of analytic steps was set, and the mechanical data on atlantoaxial horizontal dislocation-reduction and vertical dislocation-reduction were satisfied. Thus, the two surgical schemes were acceptable. The best surgical planning for the application of pulling and reducing the occipital cervical internal fixation system for treating complicated BI-AAD in patients with a complex malformation in the craniocervical junction region was studied and verified. According to the mechanical analysis during the above reduction process, a modest distraction between atlantoaxial sites before reduction and release of the joint process can reduce the obstacles of joint processes during the reduction process.

With the development of computer techniques and a finite element theory, the finite element technique has been widely applied in the field of cervical spine biomechanical research. The finite element cervical spine model not only simulates the complex mechanical system quite well, it obtains domain-wide information. In addition, the FEM complements the human in vitro experimental model.

There are some limitations of using a cervical spine FEM in this study. For example, the role of the muscle was not considered, and some tissue material parameters were obtained from the foreign literature, so they may differ from the actual values in this population. Complete simulation of the human body obviously cannot be attained, and the model should be compared and verified with some biomechanical experimental results.

## Conclusions

The study established that a 3D FEM of cervical vertebrae (C0–C4) of congenital BI-AAD patients verified the feasibility and effectiveness of the CDR technique for AAD reduction surgery. We suggest that our reduction optimization scheme could be helpful in patients with abnormal lateral atlantoaxial articulation ossification associated with congenital BI-AAD. This study provides theoretical biomechanical basis to improve the reliability of the posterior reduction surgery technique and to simplify the surgical treatment of complicated BI-AAD.

## Abbreviations

BI: basilar invagination
AAD: atlantoaxial dislocation
CDR: compression-distraction reduction
FEM: finite element model
3D: three-dimensional
COP: compression occipital plate
LAA: lateral atlantoaxial articulation
CT: computed tomography
DOF: degrees of freedom
